# Twentieth Annual Meeting of the Mississippi Valley Association of Dental Surgeons

**Published:** 1864-04

**Authors:** 


### TWENTIETH ANNUAL MEETING OF THE MISSISSIPPI VALLEY ASSOCIATION OF DENTAL SURGEONS.

### DISCUSSIONS.

1. *Anæsthetics.* — Dr. McKellops was among the early users of nitrous oxyd. In one of his early cases, the patient was short and plethoric, and the result was not quite as he desired; but the patient said he did not feel the operation. Another case—a lady had two teeth to extract; and he had just time to extract them, before consciousness returned. There was no pain. In one hundred and fifty cases, young, old and middle aged, no bad effects were observable. In one case he had taken out six teeth at one inhalation. Patients who have tried the oxyd, prefer it to ether or chloroform. He found it troublesome to keep the gas on hand, and thought it acted better when freshly prepared, or about an hour after its preparation. The gas can be prepared, in desirable quantity, in twenty minutes. In his first apparatus, he used a whisky barrel as a receiver, but now has a rubber bag in which he can preserve the gas longer. When the gas is just prepared, it irritates the throat, causing cough, if inhaled. This irritating principle is removed by keeping it in contact with water. If the patient is calm when he begins the inhalation, he usually remains calm throughout the operation. He had administered the gas three times, in rapid succession, to the same patient, with satisfactory results. A four-gallon gas-bag would be usually found sufficient for three inhalations. He had found women more patient under its influence than men.

As to chloroform, he used to give it to almost everybody, and had no bad results in his practice. A fatal case had occurred in a dispensary in which he sometimes operated. He preferred the Scotch chloroform, and was in the habit of giving an alcoholic stimulant before administering the anæsthetic. He thought the anæsthetic influence was thus more promptly produced, was better and safer, and was followed by a quicker return to consciousness. He would not give ether to a plethoric patient, nor chloroform to one far gone in consumption; and when there is an unnatural action of the heart, he would give none of these things.

Dr. Taylor does not use anæsthetics as frequently as formerly, and is very cautious in their administration. He prefers the chloric ether—a mixture of chloroform and sulphuric ether. He thought the influence of these agents was injurious to the operator; and he would greatly prefer to operate without any of them. He had not used the nitrous oxyd—thought it too cumbersome.

Dr. H. A. Smith regarded nitrous oxyd as always the same, and believed it would always produce similar effects under like circumstances. The impression upon the mind did much to rule the patient, when under the influence of anæsthetics.

Dr. Berry had not used anæsthetics much—had generally opposed their use. Had had one bad case—the patient was nearly killed. In another case, a corpulent lady insisted on taking ether, and he gave it. About ten days afterward she nearly died from consumption or something like it. She recovered, however. In another case, a nervous lady took brandy, he took out her teeth, she got very drunk, and he gave her coffee as a remedy for the drunkenness.

Dr. Taylor thought, with Dr. Smith, that the state of the mind had much influence over the patient, in modifying the action of anæsthetics.

Dr. H. A. Smith explained his previous remarks about the uniformity of action of nitrous oxyd. Impurities might be commingled with the gas; and these might act on the patient; but that was no evidence that the oxyd is not uniform in its action in like circumstances.

Dr. Taft had always felt a great interest in the subject of anæsthesia, regarded it a very important subject, one that should be very thoroughly studied by every member of the profession; thought we should endeavor to know more of their mode of action. Has some apprehension of ultimate injury to the patient and operator both, in the use of chloroform and ether. The peculiar effects of these agents, seem to be controlled, in many cases, by an undefinable, and hidden idiosyncrasy; death is many a time produced in the administration of chloroform, in apparently the most healthy and robust, while enfeebled, nervous, and even extensively diseased persons, will inhale both chloroform and ether, with apparent impunity. He thought death may be produced by chloroform in two or three ways. He regarded nitrous oxyd as very different in its action, from chloroform or ether; liked nitrous oxyd, because the operator is wholly free from its influence; because it operates more promptly upon the patient; because the patient is more quiet under its influence; and because the recovery from its influence is more sudden and complete; the patient on arousing is in a most pleasing condition, delighted with the effects of the gas, and feels better than before taking it. He considered the mode of administering in some respects objectionable, but hopes and believes it will soon be remedied. Thought it would hardly come into general use in the profession under present circumstances.

Dr. Leslie said that one advantage in using the nitrous oxyd is, that the operator inhales none of the anæsthetic. He thought it would be well to inquire what precautions are to be observed in its administration. It might not be always safe to repeat the inhalations immediately. And there might be inconvenience and perhaps danger from inhaling blood.

Dr. Taylor remarked that, when the gas in the same bag is used a second or third time, it is not pure nitrous oxyd after the first inhalation. He would not like to repeat the inhalation till the patient was done spitting, lest the blood from the first operation might produce strangulation. He thought the stomach should be empty when anæsthetics are given.

Dr. Chesebrough said that the mind usually follows the impression it receives at the beginning of the inhalations. By using the gas-bag too often without replenishing, he had seen suffocation result before anæsthesia. He thought that in nitrous oxyd, the elements were free, and that the carbonic acid from respiration had no bad effect, as long as nitrous oxyd was present.

Dr. McKellops said he had administered the gas to three different patients from the same bag, without renewing; and the effects were natural in all. He again called attention to the importance of giving alcoholic stimulants before administering ether or chloroform.

Dr. Watt said it was very important for dentists to give this subject a thorough investigation. Anæsthesia was discovered by a dentist; and dentists are now the special investigators of the action of the first anæsthetic used,—the protoxyd of nitrogen, or nitrous oxyd. It was a mistake to regard the elements composing this gas as free. It is a well recognized chemical compound. It supported combustion almost like pure oxygen, because combustible matter heated to redness was able rapidly to decompose it, and thus receive the oxygen in its nascent state. Its action in respiration was not analagous to that of condensed air, as would be the case if its elements were free—if it were merely a mixture and not a combination. He had never used the protoxyd, but was preparing to do so. He thought it important to pay great attention to its purity, and would not like to use the same bag for successive inhalations, without emptying and replenishing it. In answer to a question as to its ability to supply oxygen to the system in respiration, as atmospheric air does, he said that it undoubtedly supported respiration longer than either ether or chloroform; but the system could only get oxygen by decomposing the gas, which the carbon and hydrogen in the blood could effect only to a limited extent. True it contained more oxygen than atmospheric air; but the same is true of binoxyd of nitrogen and carbonic acid, and it was well known that the respiration of either of these is instantly fatal.

He was in the habit of using chloroform and ether—had quit *mixing* them. He preferred chloroform for mouth operations, but regarded ether as safer, especially in the hands of the inexperienced. He had never any serious consequences, but might have had a fatal case yesterday. The patient became strangled by a clot of blood. Respiration was entirely cut off. (See editorial in February number of the "Register.")

EVENING SESSION—FIRST DAY.

Dr. Collins would approve of giving alcoholic stimulants before chloroform and ether. The effects of the two classes of agents are opposite. The one counteracts the other. When the heart and arteries are small, and the pulse consequently feeble, he would not like to give chloroform.

Dr. McCullom regarded alcohol as an anæsthetic and gave cases in proof of his position.

Dr. Berry had doubts about the propriety of using anæsthetics. Thought they always hurt the patient more or less. He used alcohol, and it didn't do the patient any good, but answered his purpose.

Dr. Shadoan, in view of the great importance of this subject, connected with the fact that so much is expected from the dental profession in regard to it, suggested that this Association offer a prize for the best approved essay on the subject of anæsthesia.

Dr. H. R. Smith was opposed to offering a prize, by way of hiring dentists to kill people—to experiment on known poisons.

Dr. Shadoan inquired how long we had been "hammering" on the subject in this same way. If the anæsthetics are known poisons, we have the more need to have clear ideas about them.

Dr. H. A. Smith thought it would be an insult to scientific men to offer a prize of one hundred dollars, as had been suggested.

Dr. Taft thought a prize on this subject perfectly legitimate. This association had repeatedly awarded prizes.

Dr. H. A. Smith agreed that a prize was legitimate, and that it would be quite an honor to take it.

Dr. H. R. Smith gives anæsthetics, does not advise their use. He regards them as a curse.

(This reporter was unavoidably absent during a considerable portion of the discussions on the subject, and is, therefore unable to do justice to a number of the speakers. He hopes they will forgive him.)

*Filling Teeth, etc.* — Dr. McCullom, Chairman of the Executive Committee, explained that, as the subject of filling teeth had occupied so much time at former meetings, a principal object of the committee in proposing it now was, to have the views of members on "Wood's Plastic Metallic Filling."

Dr. Watt said he liked the plastic filling better than any other cheap material—liked it all better than the length of its name. Wished everybody would call it "Wood's Alloy." Of course it was not equal to gold; but he had found it better than tin; and he hoped all who are *determined* to use amalgams, would substitute this alloy. He would not place it in proximity to gold, on account of galvanic action that might result. He never used amalgams. Used gold almost exclusively—altogether so for seven years. But he thought somebody had to,—at least would use cheap fillings; and, of the various cheap materials, he regarded this as by far the best. The alloy should be introduced in a plastic state; and Dr. Wood's instruments, or something like them, were essential to good operations. He had not seen very decided chemical action on the alloy in the mouth, and would like to know if others had, as his observations had not been extensive.

Dr. Taft liked the alloy for a cheap filling. As to chemical action, he had noticed a yellow powder in the bottom of a cavity, where the alloy had been used over an exposed pulp, which subsequently died.

Dr. Collins had used the alloy over a year, and had observed no discoloration from chemical action. When the cavity is shallow, there must be good retaining points, as for gold. The cavity *must be dry;* and all parts of the operation should be performed with special care. It should rank next to gold.

Dr. Shadoan reported a number of cases in which he had used it, once in a very shallow cavity; and he was favorably impressed in regard to it.

MORNING SESSSON—SECOND DAY.

Dr. Taft had, at the suggestion of Dr. S. S. White, used the fluid extract of aconite, for sensitive dentine, with very decided success. He leaves the extract in the cavity three or four minutes.

Dr. Collins uses it for inflammation of the pulp.

The President elect, Dr. Berry, was here called to the chair, on taking which, he offered a few interesting and highly appropriate remarks. Said his feelings carried him back to the organization of this Association, some twenty years ago. He was gratified to be even a member of such a body, very thankful for the privilege of attending this meeting, and especially thankful for the honor now conferred on him. He thought this Association had done a little more to advance dental science than any other. It had existed longer than any other, and was still in the vigor of youth, though some of its members were becoming a little antiquated in appearance. He would endeavor to discharge the duties of the office faithfully, and would solicit the coöperation of members.

AFTERNOON SESSION.

Dr. Taylor had not used Wood's metal. Thought it was objectionable, on the same grounds as the amalgams. He wished there were no amalgams, although he used them.

He regarded tin foil as next to gold; and this was a growing feeling with him.

Dr. McKellops was opposed to the use of any amalgams. If a piece of work can be done at all, it is worth while to do it properly. He could fill any frail tooth with gold foil, if it could be filled with paste, amalgam, or Wood's alloy. Men of position and influence in the profession should be careful about endorsing any thing unworthy. Hundreds had used, and still use amalgams, because Dr. Townsend did. Dr. McK. described his mode of filling. He was a convert to the use of the mallet. He had some peculiar views: when there were approximate decays in the bicuspids, he extracted one of the teeth. If bicuspid and first molar were decayed, he would adopt the same course, especially if the teeth were crowded. He used No. 4 foil. The best material for polishing plugs (or plate) is an article called "diamond dust," which is found on the farm of Charles Hunt, near St. Louis. (Will Dr. Leslie get some of it and send it to this reporter?) In approximate cavities of molars and bicuspids, he does not "build out."

Dr. Taft said he had always fought against amalgams, and always expected to. Used nothing but gold, except experimentally. Using these cheap materials unfits us for using gold. He uses only No. 4 foil,—when he changes numbers, or manufacturers, he has difficulty.

Dr. Watt said it was all very well for dentists established in large cities to use only gold. Even in a country town, he had used no other metal for seven years. But in every community there are persons who must have cheap dentistry or none. If they must have it, somebody must do it. In the cities a man can easily decline such work, and let it go to those who do it. But in the village, with but one dentist, the case is different. Humanity has claims on him; and, if there is good cheap material, it ought to be used for those who can afford nothing better. He regarded

Wood's alloy as decidedly different from amalgams. His views of the latter were well known.

Dr. Berry thought we were not ready to take the position that no metal but gold must be used. He had several amalgam plugs in his own mouth. He would insist that *some dentists must* have something besides gold. He gave a case: A Cincinnati dentist declined to fill a tooth. A medical student, about a quarter dentist, filled it with amalgam. The tooth was comfortable and serviceable sixteen years afterward. He thought it wouldn't have lasted more than ten, if filled with gold.

Dr. Talbert thought we ought to, and at reasonable prices from those able to pay, we could afford to discriminate in favor of the poor. If a tooth was worth filling at all, it was worth filling with gold.

Dr. Foote said the subject of materials should be looked at from a medical standpoint. It ought to be remembered that amalgam plugs are about one-third mercury, which is an objectionable metal. He compared this quantity with the quantities in the various compounds of mercury, as evidence that deleterious effects might result from such plugs.

Dr. Chesebrough reported the case of a gentleman who had fourteen or fifteen amalgam plugs in his mouth, which he wore about three years. In a week after they were inserted, he experienced a tingling of the nerves, which was apparently cured by a removal of the amalgams. Six weeks after the removal, the cavities were filled with gold. In six months the tingling returned, and was better and worse, for a year or two, when the patient died.

### EVENING SESSION.

*The office — its furniture and fixtures.* — Dr. McKellops uses, and prefers Harris' iron chair. He likes the Perkins' chair, but thinks this is better. He described his instrument case, which is very convenient, but a little hard to describe.

Its form is octagonal, and it revolves on a pedestal, something like a piano stool. Each side of the octagon closes by a door, and when the door opens, it exposes a tier of drawers, adapted to the holding of foil, instruments, etc. The forceps are fitted in a revolving tray, immediately beneath the lid of the case.

Dr. H. R. Smith described his mode of saving the gold which is usually wasted through the spittoon. We failed to get his idea with sufficient accuracy to describe it.

Dr. Taylor detailed his mode, and said he had taken gold, to the value of twenty-five dollars, from the apparatus at one time.

Dr. Chesebrough uses an iron box, with mercury in the bottom of it, and water over the mercury. The mercury arrests the gold, and the water which rises above the orifice of the escape pipe, shuts off offensive gases. Dr. C. gave, on the blackboard, a diagram of his office, which is exceedingly well arranged.

Dr. Taft spoke of the great importance of having instruments arranged in perfect order.

### THIRD DAY—AFTERNOON.

*Pivot Teeth.*—Dr. Taylor was friendly to pivot teeth. For the six upper front teeth, when the roots are sound, and the artificial crowns are properly mounted, there is nothing equal to pivot teeth. When there are no other teeth to antagonize, they do not do well. He would now give one hundred dollars a root for three sound, healthy roots. By using pivots, we avoid clasps. He had seen a pivot tooth, made of hippopotamus ivory, last eighteen years.

Dr. Collins condemned wooden pivots. He inserts a tube of gold in the root; and the pivot is made of an open sided tube, with a wooden pivot enclosed in it.

Dr. Berry regarded pivoting as an unpleasant operation. A pivot tooth is always offensive. Nevertheless, he liked pivot teeth, and wore one twenty-five years. He used a gold wire in the centre of a wood pivot.

Dr. McCullom had inserted but one or two pivot teeth in the last year.

Dr. Shadoan uses osteoplastic around the pivot.

Dr. Taft liked the compressed wooden pivot better than any other. By saturating it with creosote, or gutta percha and chloroform, it would be less offensive, and would last longer.

*Miscellaneous Subjects.*—Dr. Shadoan described his mode of fastening teeth on vulcanite plates with Wood's alloy. He makes a dovetail mortise opposite to the rivets, fills it with the alloy, and places fragments of the latter around the rivets. He warms the plate and the teeth, and presses the latter to their place. When the alloy cools, it holds them firmly. A hole in a vulcanite plate can be closed in the same way.

Dr. McCullum called the attention of the profession to an automatic mallet, which could be seen at Dr. Brown's Dental depot.

Drs. Taft, McKellops, Keely and others made some general remarks on the malleting process.

Dr. Talbert read a lively and sensible essay on the duty of the profession in regard to students.

Dr. Berry, the President, made some closing remarks, and the Association adjourned.

We are sorry so good a meeting can have no better report of its proceedings. This report has been gotten up under great difficulty, and is, consequently, very imperfect. Still we believe the profession, and especially members of the association, will thank us for snatching even this much from oblivion. As no reporter was provided, we felt at liberty to try our hand. In conclusion, we can only say that, as usual, the most profitable parts of the proceedings can not be reported, hence, those who did'nt attend must forever go unblessed with a knowledge of them. W.

